# Supplementary material for: Extended KAFR: a kinematic-adaptive paradigm for the efficient analysis of surgical video
Source: NPJ Digit Surg. 2026 Oct 7;1(1):25. doi: 10.1038/s44484-026-00022-6 (PMC13645624; doi:10.1038/s44484-026-00022-6)
Supplement: Supplementary file 1 [file 44484_2026_22_MOESM1_ESM.pdf]

Supplementary Information

Extended KAFR: A kinematic-adaptive paradigm for the efficient analysis of surgical video

	method	portion	w	no relax Acc	no relax Precision	no relax Recall	no relax F1	relax Acc	relax Precision	relax Recall	relax F1
			31	84.64	79	86	82.01	87.49	85	91	87.54
			63	85.46	81	86	83.05	88.4	87	91	88.69
		0.5	127	85.28	80	83	81.08	88.97	88	90	88.51
			31	87.41	80	87	82.92	90.23	86	92	88.35
			63	88.27	82	87	84.36	91.12	88	92	89.91
		0.3	127	87.82	81	84	82.11	91.58	89	89	89.98
			31	87.25	81	87	83.52	90.1	87	91	88.81
			63	87.97	83	88	84.93	91.04	89	92	90.49
		0.1	**127**	**87.78**	**82**	**85**	**82.9**	**91.86**	**90**	**91**	**90.77**
			31	85.99	82	86	82.55	89.04	85	91	87.77
			63	87	82	87	83.79	90.07	87	91	89.22
	adaptive 1	0.05	127	86.54	81	84	82.05	90.49	89	90	89.29
			31	86.73	81	87	83.76	89.33	87	91	88.98
			63	87.67	83	87	84.88	90.4	89	92	90.23
s1	adaptive 2	0.1	127	87.36	83	84	83.19	90.83	90	90	90.23
			31	86.46	80	86	82.66	89.34	86	91	88.29
			63	87.25	82	86	83.7	90.22	88	91	89.48
		0.5	127	86.78	81	83	81.35	90.4	89	89	88.79
			31	88.02	83	87	84.65	90.75	89	91	90.25
			63	88.74	84	86	85.39	91.53	91	91	91.14
		0.4	127	88.74	84	84	83.9	92.01	92	90	90.98
			31	86.44	80	86	82.49	89.25	86	91	87.78
			63	87.35	82	86	83.89	90.16	88	91	89.14
		0.3	127	87.04	81	83	81.82	90.76	89	90	89.35
			31	85.5	80	86	82.29	88.54	86	91	88.03
			63	86.45	82	87	83.94	89.55	88	92	89.73
s2	adaptive 1	0.2	127	85.7	81	84	81.79	89.59	89	90	89.37

	method	portion	w	no relax Acc	no relax Precision	no relax Recall	no relax F1	relax Acc	relax Precision	relax Recall	relax F1
			31	86.36	80	86	82.43	89.35	86	91	88.23
			63	87.09	81	86	83.22	90.13	87	91	89.16
		0.5	127	86.28	80	83	80.78	90.19	88	90	88.57
			31	85.71	79	87	81.83	88.91	85	92	87.49
			63	87.13	81	88	83.9	90.26	87	92	89.28
		0.3	127	86.9	81	85	82.01	90.84	88	91	89.07
			31	85.92	81	85	82.36	88.95	86	90	87.94
			63	86.7	83	86	83.61	89.83	89	91	89.2
		0.1	**127**	**85.94**	**82**	**82**	**81.01**	**89.81**	**88**	**89**	**88.38**
			31	85.85	79	84	81.31	88.72	85	89	86.89
			63	86.63	81	84	82.29	89.6	87	90	88.18
	adaptive 1	0.05	127	86.4	80	82	80.45	90.24	88	89	88.44
			31	86.23	80	86	82.77	89.26	86	91	86.41
			63	86.8	82	87	83.56	89.98	88	92	89.48
s1	adaptive 2	0.1	127	86.71	81	84	81.9	90.71	89	91	89.65
			31	87.47	83	81	81.17	90.3	89	89	88.95
			63	88.26	85	81	81.91	91.06	91	89	89.87
		0.5	127	87.77	84	78	79.15	91.5	92	88	89.83
			31	87.21	83	87	84.67	89.75	89	91	89.95
			63	87.8	84	87	85.27	90.42	91	91	90.79
		0.4	127	87.51	84	84	83.54	90.75	92	90	90.68
			31	87.08	80	86	82.95	89.93	87	91	88.66
			63	87.84	82	87	84.12	90.8	88	92	89.98
		0.3	127	87.45	81	84	82.18	91.17	89	91	89.78
			31	84.86	80	86	82.21	87.76	80	86	87.5
			63	85.76	82	87	83.69	88.71	87	91	88.94
s2	adaptive 1	0.2	127	85.48	81	82	81.01	89.49	88	90	88.85

				no relax				relax			
	method	portion	w	Acc	Precision	Recall	F1	Acc	Precision	Recall	F1
			31	84.64	79	86	82.01	87.49	85	91	87.54
			63	85.46	81	86	83.05	88.4	87	91	88.69
			127	85.28	80	83	81.08	88.97	88	90	88.51
		0.5	best	85.52	80.76	84.7	82.3	88.85	87.53	90.59	88.85
			31	87.41	80	87	82.92	90.23	86	92	88.35
			63	88.27	82	87	84.36	91.12	88	92	89.91
			127	87.82	81	84	82.11	91.58	89	89	89.98
		0.3	best	88.36	81.88	85.82	83.57	91.69	89.06	91.95	90.41
			31	87.25	81	87	83.52	90.1	87	91	88.81
			63	87.97	83	88	84.93	91.04	89	92	90.49
			**127**	**87.78**	**82**	**85**	**82.9**	**91.86**	**90**	**91**	**90.77**
		0.1	**best**	**88.29**	**82.86**	**87.48**	**84.89**	**91.71**	**90.04**	**92.64**	**91.26**
			31	85.99	82	86	82.55	89.04	85	91	87.77
			63	87	82	87	83.79	90.07	87	91	89.22
			127	86.54	81	84	82.05	90.49	89	90	89.29
	adaptive 1	0.05	best	87.15	82.01	86.56	83.81	90.43	88.17	91.27	89.58
			31	86.73	81	87	83.76	89.33	87	91	88.98
			63	87.67	83	87	84.88	90.4	89	92	90.23
			127	87.36	83	84	83.19	90.83	90	90	90.23
s1	adaptive 2	0.1	best	87.78	83.5	86.08	84.56	90.92	90.52	91.29	90.81
			31	86.46	80	86	82.66	89.34	86	91	88.29
			63	87.25	82	86	83.7	90.22	88	91	89.48
			127	86.78	81	83	81.35	90.4	89	89	88.79
		0.5	best	87.59	82.21	85.89	83.78	90.69	89.08	90.99	89.94
			31	88.02	83	87	84.65	90.75	89	91	90.25
			63	88.74	84	86	85.39	91.53	91	91	91.14
		0.4	127	88.74	84	84	83.9	92.01	92	90	90.98
			best	89.11	85.1	86.76	85.84	91.92	91.68	91.67	91.64
			31	86.44	80	86	82.49	89.25	86	91	87.78
			63	87.35	82	86	83.89	90.16	88	91	89.14
			127	87.04	81	83	81.82	90.76	89	90	89.35
		0.3	best	87.54	82.31	85.92	83.84	90.64	88.48	91.23	89.74
			31	85.5	80	86	82.29	88.54	86	91	88.03
			63	86.45	82	87	83.94	89.55	88	92	89.73
			127	85.7	81	84	81.79	89.59	89	90	89.37
		0.2	best	86.69	82.09	86.73	84.04	89.92	88.72	91.86	90.09
			31	85.57	78	86	81.4	88.8	84	91	87.24
			63	86.55	80	87	83.11	89.75	86	92	88.81
			127	86.99	80	84	81.37	91.11	88	91	89.28
s2	adaptive 1	0.1	best	87.06	80.32	86.14	82.55	90.74	87.49	91.88	89.36
			31	88.08	82	88	84.93	90.54	88	92	89.81
	base	1	63	88.9	84	88	85.88	91.4	90	92	90.84
			127	88.75	84	85	84.06	92.05	91	91	91.01
s-1			best	89.34	84.89	88.24	86.33	92.01	91.07	92.41	91.64
s0	zero-level		best	87.86	83.65	84.88	84.09	90.7	90.39	90.34	90.29
			31								
			63								
			127								
		1	best	0							
s3	preliminary	0.1	best	85.54	80.37	84.22	82.05	88.16	85.76	88.4	86.93

				no relax				relax			
	method	portion	w	Acc	Precision	Recall	F1	Acc	Precision	Recall	F1
			31	86.36	80	86	82.43	89.35	86	91	88.23
			63	87.09	81	86	83.22	90.13	87	91	89.16
			127	86.28	80	83	80.78	90.19	88	90	88.57
		0.5	best	87.05	80.92	85.97	83.13	90.19	87.43	91.16	89.18
			31	85.71	79	87	81.83	88.91	85	92	87.49
			63	87.13	81	88	83.9	90.26	87	92	89.28
			127	86.9	81	85	82.01	90.84	88	91	89.07
		0.3	best	87.27	81.41	87.63	83.69	90.59	87.6	92.32	89.49
			31	85.92	81	85	82.36	88.95	86	90	87.94
			63	86.7	83	86	83.61	89.83	89	91	89.2
			**127**	**85.94**	**82**	**82**	**81.01**	**89.81**	**88**	**89**	**88.38**
		0.1	**best**	**86.72**	**83.08**	**85.81**	**83.73**	**89.89**	**88.37**	**90.9**	**89.32**
			31	85.85	79	84	81.31	88.72	85	89	86.89
			63	86.63	81	84	82.29	89.6	87	90	88.18
			127	86.4	80	82	80.45	90.24	88	89	88.44
	adaptive 1	0.05	best	87.03	81.44	84.01	82.32	90.28	88.35	89.84	88.94
			31	86.23	80	86	82.77	89.26	86	91	86.41
			63	86.8	82	87	83.56	89.98	88	92	89.48
			127	86.71	81	84	81.9	90.71	89	91	89.65
s1	adaptive 2	0.1	best	86.99	81.79	84.94	82.67	90.76	88.99	91.14	89.88
			31	87.47	83	81	81.17	90.3	89	89	88.95
			63	88.26	85	81	81.91	91.06	91	89	89.87
			127	87.77	84	78	79.15	91.5	92	88	89.83
		0.5	best	88.3	85.16	80.38	81.01	92.53	92.03	88.91	90.17
			31	87.07	81	86	83.31	90.17	88	92	89.48
			63	88	83	87	84.42	90.99	89	92	90.35
		0.4	127	88.07	83	85	83.22	91.69	91	91	90.63
			best	88.4	83.7	86.86	84.97	91.43	90.3	91.8	90.96
			31	87.08	80	86	82.95	89.93	87	91	88.66
			63	87.84	82	87	84.12	90.8	88	92	89.98
			127	87.45	81	84	82.18	91.17	89	91	89.78
		0.3	best	87.96	82.19	86.72	84.2	90.95	88.85	91.68	90.12
			31	84.86	80	86	82.21	87.76	80	86	87.5
			63	85.76	82	87	83.69	88.71	87	91	88.94
			127	85.48	81	82	81.01	89.49	88	90	88.85
		0.2	best	85.93	81.79	86.42	83.49	89.05	87.64	91.3	89.16
			31	86.74	80.00	86.00	82.84	89.37	86.00	90.00	88.07
			63	87.92	83.00	87.00	84.99	90.57	89.00	92.00	90.28
			127	87.65	82.00	83.00	82.34	91.13	90.00	90.00	89.87
s2	adaptive 1	0.1	best	88.18	83.33	85.83	84.46	91.20	90.34	91.40	90.77
			31	87.63	80	87	83.31	90.56	86	92	88.64
	base	1	63	88.46	82	88	84.27	91.46	88	92	89.74
			127	88.1	81	84	82.18	91.9	89	91	89.6
			best	88.56	81.86	86.78	83.99	91.97	88.7	92.17	90.32
			best	87.99	83.55	84.53	83.76	91.23	90.91	90.42	90.57
			31								
			63								
			127								
		1	best								
s3	preliminary		best	86.63	81.07	86.55	83.05	89.29	86.11	90.78	87.99

				no relax				relax			
	method	portion	w	Acc	Precision	Recall	F1	Acc	Precision	Recall	F1
			31	85.50	79.50	86.00	82.22	88.42	85.50	91.00	87.89
			63	86.28	81.00	86.00	83.14	89.27	87.00	91.00	88.93
			127	85.78	80.00	83.00	80.93	89.58	88.00	90.00	88.54
		0.5	best	86.29	80.84	85.34	82.72	89.52	87.48	90.88	89.02
			31	86.56	79.50	87.00	82.38	89.57	85.50	92.00	87.92
			63	87.70	81.50	87.50	84.13	90.69	87.50	92.00	89.60
			127	87.36	81.00	84.50	82.06	91.21	88.50	90.00	89.53
		0.3	best	87.82	81.65	86.73	83.63	91.14	88.33	92.14	89.95
			31	86.59	81.00	86.00	82.94	89.53	86.50	90.50	88.38
			63	87.34	83.00	87.00	84.27	90.44	89.00	91.50	89.85
			**127**	86.86	82.00	83.50	81.96	90.84	89.00	90.00	89.58
		0.1	**best**	87.51	82.97	86.65	84.31	90.80	89.21	91.77	90.29
			31	85.92	80.50	85.00	81.93	88.88	85.00	90.00	87.33
			63	86.82	81.50	85.50	83.04	89.84	87.00	90.50	88.70
			127	86.47	80.50	83.00	81.25	90.37	88.50	89.50	88.87
	adaptive 1	0.05	best	87.09	81.73	85.29	83.07	90.36	88.26	90.56	89.26
			31	86.48	80.50	86.50	83.27	89.30	86.50	91.00	87.70
			63	87.24	82.50	87.00	84.22	90.19	88.50	92.00	89.86
			127	87.04	82.00	84.00	82.55	90.77	89.50	90.50	89.94
s1	adaptive 2	0.1	best	87.39	82.65	85.51	83.62	90.84	89.76	91.22	90.35
			31	86.97	81.50	83.50	81.92	89.82	87.50	90.00	88.62
			63	87.76	83.50	83.50	82.81	90.64	89.50	90.00	89.68
			127	87.28	82.50	80.50	80.25	90.95	90.50	88.50	89.31
		0.5	best	87.95	83.69	83.14	82.40	91.61	90.56	89.95	90.06
			31	87.55	82.00	86.50	83.98	90.46	88.50	91.50	89.87
			63	88.37	83.50	86.50	84.91	91.26	90.00	91.50	90.75
		0.4	127	88.41	83.50	84.50	83.56	91.85	91.50	90.50	90.81
			best	88.76	84.40	86.81	85.41	91.68	90.99	91.74	**91.30**
			31	86.76	80.00	86.00	82.72	89.59	86.50	91.00	88.22
			63	87.60	82.00	86.50	84.01	90.48	88.00	91.50	89.56
			127	87.25	81.00	83.50	82.00	90.97	89.00	90.50	89.57
		0.3	best	87.75	82.25	86.32	84.02	90.80	88.67	91.46	89.93
			31	85.18	80.00	86.00	82.25	88.15	83.00	88.50	87.77
			63	86.11	82.00	87.00	83.82	89.13	87.50	91.50	89.34
			127	85.59	81.00	83.00	81.40	89.54	88.50	90.00	89.11
		0.2	best	86.31	81.94	86.58	83.77	89.49	88.18	91.58	89.63
			31	86.16	79.00	86.00	82.12	89.09	85.00	90.50	87.66
			63	87.24	81.50	87.00	84.05	90.16	87.50	92.00	89.55
			127	87.32	81.00	83.50	81.86	91.12	89.00	90.50	89.58
s2	adaptive 1	0.1	best	87.62	81.83	85.99	83.51	90.97	88.92	91.64	90.07
			31	87.86	81.00	87.50	84.12	90.55	87.00	92.00	89.23
	base	1	63	88.68	83.00	88.00	85.08	91.43	89.00	92.00	90.29
			127	88.43	82.50	84.50	83.12	91.98	90.00	91.00	90.31
			best	88.95	83.38	87.51	85.16	91.99	89.89	92.29	**90.98**
			best	87.93	83.60	84.71	83.93	90.97	90.65	90.38	90.43

				no relax				relax			
	method	portion	w	Acc	Precision	Recall	F1	Acc	Precision	Recall	F1
			31	1.22	0.71	0.00	0.30	1.32	0.71	0.00	0.49
			63	1.15	0.00	0.00	0.12	1.22	0.00	0.00	0.33
			127	0.71	0.00	0.00	0.21	0.86	0.00	0.00	0.04
		0.5	best	1.08	0.11	0.90	0.59	0.95	0.07	0.40	0.23
			31	1.20	0.71	0.00	0.77	0.93	0.71	0.00	0.61
			63	0.81	0.71	0.71	0.33	0.61	0.71	0.00	0.45
			127	0.65	0.00	0.71	0.07	0.52	0.71	1.41	0.64
		0.3	best	0.77	0.33	1.28	0.08	0.78	1.03	0.26	0.65
			31	0.94	0.00	1.41	0.82	0.81	0.71	0.71	0.62
			63	0.90	0.00	1.41	0.93	0.86	0.00	0.71	0.91
			**127**	1.30	0.00	2.12	1.34	1.45	1.41	1.41	1.69
		0.1	**best**	1.11	0.16	1.18	0.82	1.29	1.18	1.23	1.37
			31	0.10	2.12	1.41	0.88	0.23	0.00	1.41	0.62
			63	0.26	0.71	2.12	1.06	0.33	0.00	0.71	0.74
			127	0.10	0.71	1.41	1.13	0.18	0.71	0.71	0.60
	adaptive 1	0.05	best	0.08	0.40	1.80	1.05	0.11	0.13	1.01	0.45
			31	0.35	0.71	0.71	0.70	0.05	0.71	0.00	1.82
			63	0.62	0.71	0.00	0.93	0.30	0.71	0.00	0.53
			127	0.46	1.41	0.00	0.91	0.08	0.71	0.71	0.41
s1	adaptive 2	0.1	best	0.56	1.21	0.81	1.34	0.11	1.08	0.11	0.66
			31	0.71	2.12	3.54	1.05	0.68	2.12	1.41	0.47
			63	0.71	2.12	3.54	1.27	0.59	2.12	1.41	0.28
			127	0.70	2.12	3.54	1.56	0.78	2.12	0.71	0.74
		0.5	best	0.50	2.09	3.90	1.96	1.30	2.09	1.47	0.16
			31	0.67	1.41	0.71	0.95	0.41	0.71	0.71	0.54
			63	0.52	0.71	0.71	0.69	0.38	1.41	0.71	0.56
		0.4	127	0.47	0.71	0.71	0.48	0.23	0.71	0.71	0.25
			best	0.50	0.99	0.07	0.62	0.35	0.98	0.09	0.48
			31	0.45	0.00	0.00	0.33	0.48	0.71	0.00	0.62
			63	0.35	0.00	0.71	0.16	0.45	0.00	0.71	0.59
			127	0.29	0.00	0.71	0.25	0.29	0.00	0.71	0.30
		0.3	best	0.30	0.08	0.57	0.25	0.22	0.26	0.32	0.27
			31	0.45	0.00	0.00	0.06	0.55	4.24	3.54	0.37
			63	0.49	0.00	0.00	0.18	0.59	0.71	0.71	0.56
			127	0.16	0.00	1.41	0.55	0.07	0.71	0.00	0.37
		0.2	best	0.54	0.21	0.22	0.39	0.62	0.76	0.40	0.66
			31	0.83	1.41	0.00	1.02	0.40	1.41	0.71	0.59
			63	0.97	2.12	0.00	1.33	0.58	2.12	0.00	1.04
			127	0.47	1.41	0.71	0.69	0.01	1.41	0.71	0.42
s2	adaptive 1	0.1	best	0.79	2.13	0.22	1.35	0.33	2.02	0.34	1.00
			31	0.32	1.41	0.71	1.15	0.01	1.41	0.00	0.83
	base	1	63	0.31	1.41	0.00	1.14	0.04	1.41	0.00	0.78
			127	0.46	2.12	0.71	1.33	0.11	1.41	0.00	1.00
			best	0.55	2.14	1.03	1.65	0.03	1.68	0.17	0.93
			best	0.09	0.07	0.25	0.23	0.37	0.37	0.06	0.20

name	time (s)
train	23'42''
train_0_1	8'12''
train_0_2	10'24''
train_0_3	12'329
train_0_4	
train_0_5	

				no relax				relax				
	method	portion	w	Acc	Precision	Recall	F1	Acc	Precision	Recall	F1	
		0.3	best	86.69	78.93	83.69	80.60	91.17	87.81	91.68	89.38	
		0.1	best	85.92	83.11	82.17	82.00	89.30	89.74	89.48	89.36	
	Active 1	0.05	best	86.20	80.26	85.11	82.27	89.09	86.24	89.77	87.87	v1
s1	Active 2	0.1	best	87.4	82.56	83.17	82.58	91.01	90.72	89.71	90.13	
		0.4	best	86.82	83.66	83.95	83.43	90.01	91.00	89.94	90.29	
		0.3	best	87.98	83.71	85.97	84.54	91.17	90.61	91.35	90.90	v1_2
		0.2	best	88.31	83.31	85.56	84.19	91.55	90.3	90.79	90.52	
	Active 1	0.1	best	85.60	80.95	84.25	81.80	89.36	88.14	90.22	88.91	
		0.4	best	88.03	83.08	86.53	84.54	91.03	89.67	91.48	90.49	
s2	Activ 2	0.3	best	87.16	83.8	84.62	83.91	90.11	90.27	90.57	90.3	
		0.2	best	87.41	82.97	87	84.64	90.4	89.3	91.64	90.36	

				no relax				relax			
	method	portion		Acc	Precision	Recall	F1	Acc	Precision	Recall	F1
		0.3	best	87.08	83.21	84.79	83.83	89.85	89.37	90.09	89.59
		0.1	best	87.58	83.93	84.52	84.03	89.89	88.99	88.82	88.82
	Active 1	0.05	best	87.23	82.45	84.27	82.71	90.38	89.03	89.87	89.32
s1	Active 2	0.1	best	87.34	82.06	84.41	82.69	90.98	89.58	90.99	90.19
		0.4	best	89.34	84.42	86	85	92.1	90.91	91.01	90.88
		0.3	best	88.37	84.46	85.91	85.06	91.17	90.97	91.07	90.97
		0.2	best	87.79	83.48	86.37	84.74	90.62	90.33	91.2	90.7
	Active 1	0.1	best	87.82	82.81	86.51	84.26	91.08	89.79	91.84	90.70
		0.4	best	88.07	83.46	86.38	84.76	91	89.82	91.6	90.63
s2	Activ 2	0.3	best	87.48	83.08	86.48	84.53	90.57	90.11	91.51	90.72
		0.2	best	85.49	83.19	84.57	83.47	88.34	89.59	89.96	89.55

				no relax				relax			
	method	portion		Acc	Precision	Recall	F1	Acc	Precision	Recall	F1
		0.3	best	86.89	81.07	84.24	82.22	90.51	88.59	90.89	89.49
		0.1	best	86.75	83.52	83.35	83.02	89.60	89.37	89.15	89.09
	Active 1	0.05	best	86.72	81.36	84.69	82.49	89.74	87.64	89.82	88.60
s1	Active 2	0.1	best	87.37	82.31	83.79	82.64	91.00	90.15	90.35	90.16
		0.4	best	88.08	84.04	84.98	84.22	91.06	90.96	90.48	90.59
		0.3	best	88.18	84.09	85.94	84.80	91.17	90.79	91.21	90.94
		0.2	best	88.05	83.40	85.97	84.47	91.09	90.32	91.00	90.61
	Active 1	0.1	best	86.71	81.88	85.38	83.03	90.22	88.97	91.03	89.81
		0.4	best	88.05	83.27	86.46	84.65	91.02	89.75	91.54	90.56
s2	Activ 2	0.3	best	87.32	83.44	85.55	84.22	90.34	90.19	91.04	90.51
		0.2	best	86.45	83.08	85.79	84.06	89.37	89.45	90.80	89.96

	method	portion	w	no relax Acc	no relax Precision	no relax Recall	no relax F1	relax Acc	relax Precision	relax Recall	relax F1
			31	84.64	79	86	82.01	87.49	85	91	87.54
			63	85.46	81	86	83.05	88.4	87	91	88.69
			127	85.28	80	83	81.08	88.97	88	90	88.51
		0.5	best	85.52	80.76	84.7	82.3	88.85	87.53	90.59	88.85
			31	87.41	80	87	82.92	90.23	86	92	88.35
			63	88.27	82	87	84.36	91.12	88	92	89.91
			127	87.82	81	84	82.11	91.58	89	89	89.98
		0.3	best	88.36	81.88	85.82	83.57	91.69	89.06	91.95	90.41
			31	87.25	81	87	83.52	90.1	87	91	88.81
			63	87.97	83	88	84.93	91.04	89	92	90.49
			**127**	**87.78**	**82**	**85**	**82.9**	**91.86**	**90**	**91**	**90.77**
		0.1	**best**	**88.29**	**82.86**	**87.48**	**84.89**	**91.71**	**90.04**	**92.64**	**91.26**
			31	85.99	82	86	82.55	89.04	85	91	87.77
			63	87	82	87	83.79	90.07	87	91	89.22
			127	86.54	81	84	82.05	90.49	89	90	89.29
	adaptive 1	0.05	best	87.15	82.01	86.56	83.81	90.43	88.17	91.27	89.58
			31	86.73	81	87	83.76	89.33	87	91	88.98
			63	87.67	83	87	84.88	90.4	89	92	90.23
			127	87.36	83	84	83.19	90.83	90	90	90.23
s1	adaptive 2	0.1	best	87.78	83.5	86.08	84.56	90.92	90.52	91.29	90.81
			31	86.46	80	86	82.66	89.34	86	91	88.29
			63	87.25	82	86	83.7	90.22	88	91	89.48
			127	86.78	81	83	81.35	90.4	89	89	88.79
		0.5	best	87.59	82.21	85.89	83.78	90.69	89.08	90.99	89.94
			31	88.02	83	87	84.65	90.75	89	91	90.25
		0.4	63	88.74	84	86	85.39	91.53	91	91	91.14
			127	88.74	84	84	83.9	92.01	92	90	90.98
			best	89.11	85.1	86.76	85.84	91.92	91.68	91.67	91.64
			31	86.44	80	86	82.49	89.25	86	91	87.78
			63	87.35	82	86	83.89	90.16	88	91	89.14
			127	87.04	81	83	81.82	90.76	89	90	89.35
		0.3	best	87.54	82.31	85.92	83.84	90.64	88.48	91.23	89.74
			31	85.5	80	86	82.29	88.54	86	91	88.03
			63	86.45	82	87	83.94	89.55	88	92	89.73
			127	85.7	81	84	81.79	89.59	89	90	89.37
		0.2	best	86.69	82.09	86.73	84.04	89.92	88.72	91.86	90.09
			31	85.57	78	86	81.4	88.8	84	91	87.24
			63	86.55	80	87	83.11	89.75	86	92	88.81
			127	86.99	80	84	81.37	91.11	88	91	89.28
s2	adaptive 1	0.1	best	87.06	80.32	86.14	82.55	90.74	87.49	91.88	89.36
			31	88.08	82	88	84.93	90.54	88	92	89.81
	base	1	63	88.9	84	88	85.88	91.4	90	92	90.84
			127	88.75	84	85	84.06	92.05	91	91	91.01
s-1			best	89.34	84.89	88.24	86.33	92.01	91.07	92.41	91.64
s0	zero-level		best	87.86	83.65	84.88	84.09	90.7	90.39	90.34	90.29
			31								
			63								
			127								
		1	best	0							
s3	preliminary	0.1	best	85.54	80.37	84.22	82.05	88.16	85.76	88.4	86.93

	method	portion	w	no relax Acc	no relax Precision	no relax Recall	no relax F1	relax Acc	relax Precision	relax Recall	relax F1
			31	86.36	80	86	82.43	89.35	86	91	88.23
			63	87.09	81	86	83.22	90.13	87	91	89.16
			127	86.28	80	83	80.78	90.19	88	90	88.57
		0.5	best	87.05	80.92	85.97	83.13	90.19	87.43	91.16	89.18
			31	85.71	79	87	81.83	88.91	85	92	87.49
			63	87.13	81	88	83.9	90.26	87	92	89.28
			127	86.9	81	85	82.01	90.84	88	91	89.07
		0.3	best	87.27	81.41	87.63	83.69	90.59	87.6	92.32	89.49
			31	85.92	81	85	82.36	88.95	86	90	87.94
			63	86.7	83	86	83.61	89.83	89	91	89.2
			**127**	**85.94**	**82**	**82**	**81.01**	**89.81**	**88**	**89**	**88.38**
		0.1	**best**	**86.72**	**83.08**	**85.81**	**83.73**	**89.89**	**88.37**	**90.9**	**89.32**
			31	85.85	79	84	81.31	88.72	85	89	86.89
			63	86.63	81	84	82.29	89.6	87	90	88.18
			127	86.4	80	82	80.45	90.24	88	89	88.44
	adaptive 1	0.05	best	87.03	81.44	84.01	82.32	90.28	88.35	89.84	88.94
			31	86.23	80	86	82.77	89.26	86	91	86.41
			63	86.8	82	87	83.56	89.98	88	92	89.48
			127	86.71	81	84	81.9	90.71	89	91	89.65
s1	adaptive 2	0.1	best	86.99	81.79	84.94	82.67	90.76	88.99	91.14	89.88
			31	87.47	83	81	81.17	90.3	89	89	88.95
			63	88.26	85	81	81.91	91.06	91	89	89.87
			127	87.77	84	78	79.15	91.5	92	88	89.83
		0.5	best	88.3	85.16	80.38	81.01	92.53	92.03	88.91	90.17
			31	87.07	81	86	83.31	90.17	88	92	89.48
		0.4	63	88	83	87	84.42	90.99	89	92	90.35
			127	88.07	83	85	83.22	91.69	91	91	90.63
			best	88.4	83.7	86.86	84.97	91.43	90.3	91.8	90.96
			31	87.08	80	86	82.95	89.93	87	91	88.66
			63	87.84	82	87	84.12	90.8	88	92	89.98
			127	87.45	81	84	82.18	91.17	89	91	89.78
		0.3	best	87.96	82.19	86.72	84.2	90.95	88.85	91.68	90.12
			31	84.86	80	86	82.21	87.76	80	86	87.5
			63	85.76	82	87	83.69	88.71	87	91	88.94
			127	85.48	81	82	81.01	89.49	88	90	88.85
		0.2	best	85.93	81.79	86.42	83.49	89.05	87.64	91.3	89.16
			31	86.74	80.00	86.00	82.84	89.37	86.00	90.00	88.07
			63	87.92	83.00	87.00	84.99	90.57	89.00	92.00	90.28
			127	87.65	82.00	83.00	82.34	91.13	90.00	90.00	89.87
s2	adaptive 1	0.1	best	88.18	83.33	85.83	84.46	91.20	90.34	91.40	90.77
			31	87.63	80	87	83.31	90.56	86	92	88.64
	base	1	63	88.46	82	88	84.27	91.46	88	92	89.74
			127	88.1	81	84	82.18	91.9	89	91	89.6
			best	88.56	81.86	86.78	83.99	91.97	88.7	92.17	90.32
			best	87.99	83.55	84.53	83.76	91.23	90.91	90.42	90.57
			31								
			63								
			127								
		1	best								
s3	preliminary		best	86.63	81.07	86.55	83.05	89.29	86.11	90.78	87.99

	method	portion	w	no relax Acc	no relax Precision	no relax Recall	no relax F1	relax Acc	relax Precision	relax Recall	relax F1
			31	85.50	79.50	86.00	82.22	88.42	85.50	91.00	87.89
			63	86.28	81.00	86.00	83.14	89.27	87.00	91.00	88.93
			127	85.78	80.00	83.00	80.93	89.58	88.00	90.00	88.54
		0.5	best	86.29	80.84	85.34	82.72	89.52	87.48	90.88	89.02
			31	86.56	79.50	87.00	82.38	89.57	85.50	92.00	87.92
			63	87.70	81.50	87.50	84.13	90.69	87.50	92.00	89.60
			127	87.36	81.00	84.50	82.06	91.21	88.50	90.00	89.53
		0.3	best	87.82	81.65	86.73	83.63	91.14	88.33	92.14	89.95
			31	86.59	81.00	86.00	82.94	89.53	86.50	90.50	88.38
			63	87.34	83.00	87.00	84.27	90.44	89.00	91.50	89.85
			**127**	86.86	82.00	83.50	81.96	90.84	89.00	90.00	89.58
		0.1	**best**	87.51	82.97	86.65	84.31	90.80	89.21	91.77	90.29
			31	85.92	80.50	85.00	81.93	88.88	85.00	90.00	87.33
			63	86.82	81.50	85.50	83.04	89.84	87.00	90.50	88.70
			127	86.47	80.50	83.00	81.25	90.37	88.50	89.50	88.87
	adaptive 1	0.05	best	87.09	81.73	85.29	83.07	90.36	88.26	90.56	89.26
			31	86.48	80.50	86.50	83.27	89.30	86.50	91.00	87.70
			63	87.24	82.50	87.00	84.22	90.19	88.50	92.00	89.86
			127	87.04	82.00	84.00	82.55	90.77	89.50	90.50	89.94
s1	adaptive 2	0.1	best	87.39	82.65	85.51	83.62	90.84	89.76	91.22	90.35
			31	86.93	81.41	83.96	82.18	89.80	87.44	90.14	88.63
			63	87.80	83.23	84.00	83.07	90.70	89.34	90.24	89.69
			127	87.26	82.13	80.81	80.54	90.97	90.13	88.73	89.27
		0.5	best	87.99	83.46	83.77	82.86	91.46	90.23	90.29	90.09
			31	87.26	81.41	85.90	83.40	90.23	87.89	91.17	89.39
		0.4	63	88.16	83.08	86.11	84.47	91.11	89.65	91.38	90.48
			127	88.00	82.65	83.56	82.72	91.60	90.81	90.18	90.33
			best	88.46	83.79	86.32	84.85	91.49	90.50	91.62	**90.99**
			31	87.18	80.73	86.33	83.28	89.94	87.03	91.15	88.71
			63	87.97	82.53	86.77	84.42	90.79	88.56	91.60	89.94
			127	87.59	81.54	83.76	82.41	91.18	89.45	90.51	89.84
		0.3	best	88.14	82.83	86.64	84.51	91.09	89.24	91.61	90.32
			31	85.92	80.41	86.00	82.58	88.79	84.25	89.33	88.07
			63	86.86	82.51	86.84	84.10	89.77	88.02	91.47	89.58
			127	86.33	81.49	82.90	81.62	90.13	88.83	89.99	89.27
		0.2	best	87.04	82.51	86.43	84.02	90.08	88.58	91.48	89.82
			31	86.37	79.90	86.22	82.58	89.37	85.86	90.88	88.20
			63	87.29	81.90	86.82	84.02	90.34	88.02	91.88	89.73
			127	87.32	81.37	83.67	82.03	91.14	89.33	90.49	89.72
s2	adaptive 1	0.1	best	87.69	82.33	86.23	83.81	91.03	89.20	91.71	90.24
			31	87.86	81.00	87.50	84.12	90.55	87.00	92.00	89.23
	base	1	63	88.68	83.00	88.00	85.08	91.43	89.00	92.00	90.29
			127	88.43	82.50	84.50	83.12	91.98	90.00	91.00	90.31
			best	88.95	83.38	87.51	85.16	91.99	89.89	92.29	**90.98**
			best	87.93	83.60	84.71	83.93	90.97	90.65	90.38	90.43

	method	portion	w	no relax Acc	no relax Precision	no relax Recall	no relax F1	relax Acc	relax Precision	relax Recall	relax F1
			31	1.22	0.71	0.00	0.30	1.32	0.71	0.00	0.49
			63	1.15	0.00	0.00	0.12	1.22	0.00	0.00	0.33
			127	0.71	0.00	0.00	0.21	0.86	0.00	0.00	0.04
		0.5	best	1.08	0.11	0.90	0.59	0.95	0.07	0.40	0.23
			31	1.20	0.71	0.00	0.77	0.93	0.71	0.00	0.61
			63	0.81	0.71	0.71	0.33	0.61	0.71	0.00	0.45
			127	0.65	0.00	0.71	0.07	0.52	0.71	1.41	0.64
		0.3	best	0.77	0.33	1.28	0.08	0.78	1.03	0.26	0.65
			31	0.94	0.00	1.41	0.82	0.81	0.71	0.71	0.62
			63	0.90	0.00	1.41	0.93	0.86	0.00	0.71	0.91
			**127**	1.30	0.00	2.12	1.34	1.45	1.41	1.41	1.69
		0.1	**best**	1.11	0.16	1.18	0.82	1.29	1.18	1.23	1.37
			31	0.10	2.12	1.41	0.88	0.23	0.00	1.41	0.62
			63	0.26	0.71	2.12	1.06	0.33	0.00	0.71	0.74
			127	0.10	0.71	1.41	1.13	0.18	0.71	0.71	0.60
	adaptive 1	0.05	best	0.08	0.40	1.80	1.05	0.11	0.13	1.01	0.45
			31	0.35	0.71	0.71	0.70	0.05	0.71	0.00	1.82
			63	0.62	0.71	0.00	0.93	0.30	0.71	0.00	0.53
			127	0.46	1.41	0.00	0.91	0.08	0.71	0.71	0.41
s1	adaptive 2	0.1	best	0.56	1.21	0.81	1.34	0.11	1.08	0.11	0.66
			31	0.51	1.51	2.62	0.88	0.48	1.50	1.03	0.33
			63	0.51	1.57	2.64	1.00	0.43	1.53	1.08	0.20
			127	0.50	1.63	2.56	1.21	0.55	1.63	0.64	0.52
		0.5	best	0.36	1.52	2.96	1.60	0.96	1.58	1.19	0.13
			31	0.69	1.43	1.16	1.20	0.49	1.16	0.76	0.90
		0.4	63	0.52	0.89	0.84	0.90	0.37	1.17	0.54	0.60
			127	0.77	1.56	1.70	1.49	0.46	1.30	0.75	0.84
			best	0.62	1.26	0.85	1.05	0.41	1.09	0.22	0.64
			31	0.80	1.26	0.58	0.99	0.70	1.05	0.25	0.95
			63	0.70	0.92	0.68	0.73	0.63	0.98	0.53	0.78
			127	0.63	0.93	0.67	0.73	0.43	0.77	0.50	0.52
		0.3	best	0.70	1.01	0.68	0.87	0.53	1.01	0.35	0.71
			31	1.32	0.70	0.01	0.58	1.18	3.70	2.89	0.59
			63	1.36	0.89	0.28	0.50	1.19	1.03	0.50	0.58
			127	1.28	0.85	1.01	0.54	1.02	0.76	0.02	0.38
		0.2	best	1.33	1.00	0.30	0.52	1.12	0.88	0.33	0.57
			31	0.70	1.85	0.38	1.07	0.57	1.80	0.82	1.04
			63	0.69	1.65	0.31	0.94	0.52	1.75	0.21	0.80
			127	0.33	1.18	0.58	0.58	0.04	1.15	0.50	0.39
s2	adaptive 1	0.1	best	0.57	1.74	0.45	1.09	0.25	1.51	0.27	0.77
			31	0.32	1.41	0.71	1.15	0.01	1.41	0.00	0.83
	base	1	63	0.31	1.41	0.00	1.14	0.04	1.41	0.00	0.78
			127	0.46	2.12	0.71	1.33	0.11	1.41	0.00	1.00
			best	0.55	2.14	1.03	1.65	0.03	1.68	0.17	0.93
			best	0.09	0.07	0.25	0.23	0.37	0.37	0.06	0.20

method	portion	w	no relax Acc	no relax Precision	no relax Recall	no relax F1	relax Acc	relax Precision	relax Recall	relax F1
		31								
		63								
		127								
	0.5	best								
		31								
	0.4	63								
		127								
		best								
		31								
		63								
		127								
	0.3	best								
		31								
		63								
		127								
	0.2	best								
		31								
		63								
		127								
adaptive 1	0.1	best								
		31	86.87	81.22	84.87	82.72	89.77	87.33	90.41	88.65
		63	87.89	82.69	84.99	83.59	90.83	89.02	90.71	89.73
		127	87.24	81.38	81.44	81.12	91.01	89.4	89.2	89.19
	0.5	best	88.07	83.02	85.03	83.78	91.16	89.57	90.96	90.15
		31	86.68	80.24	84.69	82.25	89.78	86.68	90.5	88.45
	0.4	63	87.74	82.23	85.33	83.59	90.82	88.96	91.14	89.96
		127	87.2	80.94	81.69	81.05	91.1	89.43	89.53	89.38
		best	87.87	82.58	85.34	83.75	91.11	89.52	91.38	90.37
		31	88.03	82.18	87	84.39	90.65	88.1	91.44	89.68
		63	88.73	83.59	87.31	85.26	91.41	89.69	91.79	90.69
		127	88.27	82.61	84.27	83.22	91.61	90.34	90.54	90.38
	0.3	best	88.91	84	87.28	85.49	91.68	90.38	91.93	91.11
		31	87.39	81.22	86.01	83.25	90.08	86.74	91	88.67
		63	88.38	83.54	86.52	84.66	91.06	89.05	91.4	90.07
		127	87.8	82.48	82.7	82.05	91.31	89.49	89.97	89.58
	0.2	best	88.51	83.65	86.13	84.53	91.28	89.38	91.28	90.21
		31	86.81	81.7	86.65	83.5	89.94	87.59	91.63	89.3
		63	87.39	82.71	86.47	83.95	90.7	89.07	91.63	90.11
		127	87.33	82.1	84.02	82.39	91.19	89.98	90.48	90.02
adaptive 1	0.1	best	87.84	83.34	86.71	84.41	91.14	89.76	91.84	90.59
